# Methodological concerns in the OASIS-HF study: phenotypic misclassification and selection bias

**DOI:** 10.1093/eschf/xvag039

**Published:** 2026-01-23

**Authors:** Ahmed B Shamsulddin

**Affiliations:** Al-Yarmouk Teaching Hospital, 79V2+JWG, Jinub Street, Baghdad, Iraq

**Keywords:** OASIS-HF, Phenotype, Misclassification, Selection Bias

To the Editor,

Amioka *et al*.^1^ are to be commended for the OASIS-HF study, which provides valuable insights into the use of sodium-glucose cotransporter 2 inhibitors (SGLT2i) in the ‘super-elderly’ population—a demographic often excluded from major clinical trials.

While their findings are encouraging, as they found that SGLT2 inhibitors (SGLT2i) led to a lower risk of the primary composite outcome (cardiovascular death or heart failure rehospitalization) in elderly patients than conventional therapy, a careful review of the study design reveals two critical methodological flaws that may compromise the validity of the conclusions.

*First*, the authors defined heart failure with preserved ejection fraction (HFpEF) as a left ventricular ejection fraction (LVEF) of ≥40%. This definition contradicts the established international consensus, including the 2021 ESC Guidelines and the 2022 AHA/ACC/HFSA Guidelines, which strictly define HFpEF as LVEF ≥50%.^2,3^

The range of 40%–49% is universally recognized as heart failure with mildly reduced ejection fraction (HFmrEF). As demonstrated by Lauritsen *et al*.^4^ Heart failure with mildly reduced ejection fraction is a distinct clinical entity with significantly lower mortality compared to HFpEF.

By conflating these two distinct phenotypes, the OASIS-HF study creates significant confounding. HFmrEF patients often share characteristics with HFrEF, including a more robust response to neurohormonal blockade. Consequently, the reported benefits in the ‘HFpEF’ group (≥40%) may be driven by the inclusion of the HFmrEF cohort (40%–49%), obscuring the true efficacy in the elderly ‘true HFpEF’ population (≥50%).

Second, the *post hoc* exclusion of 10 patients from the SGLT2i group specifically due to adverse events (8 with urinary tract infections and 2 with hypotension) represents a departure from intention-to-treat principles. This exclusion distinguishes the study population from real-world practice, where these specific adverse events are mechanistic drivers of treatment failure. Hypotension and infections are known mechanistic risks of SGLT2i, particularly when combined with loop diuretics in the elderly.^5^

Excluding patients who experienced these treatment failures introduces a profound selection bias, effectively ‘sanitizing’ the intervention arm to include only those who tolerated the therapy. This ‘survivorship bias’ likely exaggerates the safety profile and efficacy outcomes.

So, I urge the caution in interpretation the results of the trial simultaneously proposing that the authors provide:

A stratified analysis separating patients with HFmrEF (40%–49%) from true HFpEF (≥50%) to align with the guidelines standards.

An intention-to-treat analysis that includes the 10 patients excluded for adverse events in SGLT2i group.

Without these clarifications, the applicability of the OASIS-HF findings to clinical practice remains uncertain.

## Authors’ contributions

Ahmed B. Shamsulddin (Conceptualization [lead]; Investigation [lead]; Writing—original draft [lead]; Writing—review & editing [lead]).
